# Long-term results of implant-based functional rehabilitation of patients with ectodermal dysplasia

**DOI:** 10.1186/1746-160X-8-S1-I15

**Published:** 2012-05-25

**Authors:** Siegmar Reinert

**Affiliations:** 1 Department of Oral and Maxillofacial Surgery, University Hospital Tübingen, Germany

## 

Functional dental rehabilitation of patients with ectodermal dysplasia (ED) still remains a challenge due to severe hypodontia and underdeveloped alveolar ridges that often preclude implant placement without surgical augmentation. Only few data about technique and timing of augmentation and implant placement can be found in the literature. We have established a surgical algorithm as part of a functional dental rehabilitation concept for ED patients.

After clinical and radiographic analysis 8 ED patients with typical hypodontia and underdeveloped alveolar ridges of maxilla and mandible were treated by augmentation with autogenous iliac or mandibular bone. In one case, lateralisation of the inferior alveolar nerve was performed. Three months later implants were inserted and loaded after an interval of 6 months. Sulcus reconstruction with split skin grafts had to be done in three patients.

Since then - on average 5 years after loading - no implant has been lost. No sensory deficit has been observed. Based on our results we conclude that in the growing ED patient removable prostheses should be recommended in general, implant placement only in selected cases. When the growth phase is finished, augmentation of the mandible and/or maxilla with autogenous bone may be safely performed, followed by implant placement in a second procedure and loading of the implants. Iliac bone is most often used due to the relatively large amount of bone needed.

